# Editorial Notes, News and Miscellany

**Published:** 1913-11

**Authors:** 


### Editorial Notes, News and Miscellany.

---

Dr. E. E. Myer's paper on the "Relative Value of Turtle Tuberculin in the Treatment of Tuberculosis" is reproduced herewith to enable me to fill orders for more than one hundred copies of the October number, which contained it. It bears re-reading.

---

We are pained to announce that our "eugenics" editor, Dr. Malone Duggan, is a patient at Johns Hopkins for a surgical operation. Dr. T. Y. Hull sends splendid "copy" for the eugenics department in his absence, and we hope he will be able to resume his pen before our next issue.

---

Dr. B. M. Worsham, of El Paso, long time Superintendent of the (Austin) State Lunatic Asylum, has "thrown his hat in the ring" and will be a candidate for Governor of Texas next July. He'd make a good one, and will get a strong backing, especially from the medical profession, with which he is deservedly very popular.

---

Dr. C. H. Hughes, the distinguished founder and editor of *The Alienist and Neurologist,* has had another honor conferred upon him in addition to the many he so gracefully wears. It is no less than honorary membership in The Physio-Chemical Society of Palermo, Italy. *The Alienist and Neurologist* is constantly growing in influence and is greatly appreciated abroad as well as at home.

---

Rare Chance for a Sanitarium.—For sale, in the suburbs of Austin, overlooking the city and the beautiful Colorado valley, a handsome two-story building, ten rooms, and ten acres of ground.

This is an ideal place for a sanitarium, especially as the noted South Austin Natural Sulphur Well is on the place, together with orchard and all necessary outbuildings. In the advertising department will be found an analysis of the water. For price, terms and all details, address J. H. W. Williams, Austin, Texas, care of this journal, or the owner, Mrs. P. C. Beaty, 2215 S. 68th street, Philadelphia, Pa.

---

Medicine and law are alike in this respect, that neither can be thoroughly established. Good laws cannot be enacted on the spur of the moment; they represent the gradual growth of a social habit or custom, so that by the time the law is ready to be enacted by a legislative body, it has already become a practical law among the community. The same thing is true of a remedy. Medicine comes and medicine goes, but only those based upon logic and having proved their therapeutic efficiency by the incontrovertible evidence of clinicians continue to live and grow in popularity. Such a product is Hayden's Viburnum Compound. Its reputation rests upon a half century of actual testing at the bedside and in the physician's office, and its reliability is as dependable as that of a law which has been operating in a community for a long time.

---

The Alvarenga Prize.—The College of Physicians of Philadelphia announces that the next award of the Alvarenga Prize, being the income for one year of the bequest of the late Senor Alvarenga, and amounting to about $180, will be made on July 14, 1914, provided that an essay deemed by the committee of award to be worthy of the prize shall have been offered. Essays intended for competition may be upon any subject in medicine, but cannot have been published. They must be typewritten, and if written in a language other than English, should be accompanied by an English translation, and must be received by the secretary of the college on or before May 1, 1914. Each essay must be sent without signature, but must be plainly marked with a motto and be accompanied by a sealed envelope having on its outside the motto of the paper and within the name and address of the author. It is a condition of competition that the successful essay or a copy of it shall remain in possession of the college; other essays will be returned upon application within three months after the award. Thomas R. Neilson, M. D., Secretary, 19 South Twenty-second Street, Philadelphia.

